# 

**Published:** 1862-04

**Authors:** 


### DENTAL REGISTER

### OF THE WEST.

SMITHSONIAN INSTITUTION

EDITED BY

J. TAFT AND GEO. WATT.

APRIL,–1862.

---

**MONTHLY:**

**Three Dollars per Annum, in advance.**

---

CINCINNATI:

J. TAFT, PUBLISHER.

J. TAFT, DENTIST, NO. 56 WEST FOURTH STREET.

